# Self-Rated Depressive Symptoms in Children and Youth with and without Cerebral Palsy: A Pilot Study

**DOI:** 10.3390/bs10110167

**Published:** 2020-11-01

**Authors:** Daiki Asano, Masaki Takeda, Satoshi Nobusako, Shu Morioka

**Affiliations:** 1 Department of Rehabilitation, Japan Baptist Hospital, Kyoto 606-8273, Japan; 2 Department of Rehabilitation, Beppu Developmental Medical Center, Oita 874-0838, Japan; masaki-t@iwk.bbiq.jp; 3 Neurorehabilitation Research Center, Kio University, Nara 635-0832, Japan; s.nobusako@kio.ac.jp (S.N.); s.morioka@kio.ac.jp (S.M.)

**Keywords:** cerebral palsy, depression, mental health, behavioral problems

## Abstract

Children with cerebral palsy (CP) often exhibit mental health problems, such as depressive symptoms. The purpose of this study was to describe the self-rated depressive symptoms in children with and without CP and to investigate the associated predictors. Participants included 24 children with CP and 33 typically developing (TD) children. Depressive symptoms were assessed using the Birleson Depression Self-Rating Scale for Children. Parents of the participants completed the Strengths and Difficulties Questionnaire. Severity of self-rated depressive symptoms was higher in children with CP than that in TD children. Particularly, decline in activities and enjoyment was identified as a contributor to the increased severity of depressive symptoms. Hierarchical multiple regression analysis revealed that the greater severity of depressive symptoms in children with CP was mediated by hyperactivity/inattention and peer problems. Our study suggests that it is imperative to provide opportunities to participate in social activities from an early age.

## 1. Introduction

Cerebral palsy (CP) is one of the most common physical disabilities that develop in the early postnatal period. It affects the development of movement and posture. Children and adolescents with CP experience many activity limitations [1]. Related motor disorders are often linked to other impairments in cognition, communication, and perception [2]. Some studies have also reported elevated levels of emotional, social, and behavioral symptoms suggestive of mental health problems [3,4]. These problems may reduce the motivation to participate in various physical activities [5]. Previous research on school-age children and toddlers with motor impairments indicates that these children exhibit a lower level of task motivation than age-matched peers [6,7]. According to Thelen’s dynamic systems theory, motivation is one of several crucial components that must coalesce for a new behavior to appear [8]. Motivation to move is also a very important factor for rehabilitation approach aimed at improving motor function in children with motor impairments and motor development in non-impaired children [9,10,11]. Reduced activity levels and participation restrictions due to these impairments may lead to decline in mental health and quality of life (QOL) in children with motor impairments when compared with typically developing (TD) peers.

Although previous studies have demonstrated that children with CP or those with developmental coordination disorders often have mental health disorders [12,13,14], children with CP who can self-report showed quality of life (QOL) similar to their able-bodied peers in some studies [15,16]. Colver et al. reported that adolescents with CP had significantly lower QOL when compared with peers from the general population in only one domain (social support and peers) [17]. These conflicts may be attributed to the difference between parents-reporting and self-reporting, as parents usually underestimate their child’s QOL [5,18]. However, a number of studies conducted to investigate the prevalence of mental health disorders in children with CP rely mostly on parent-reported questionnaires such as the Strengths and Difficulties Questionnaire (SDQ) and the Child Behavior Checklist [19]. Moreover, although some studies reported that adults with CP often have depressive symptoms [20,21,22,23], very few studies have analyzed depression among children with CP. Recent investigations have demonstrated that children with CP had a higher prevalence of depression when compared with controls. However, after adjusting for sociodemographic or physical factors, children with CP did not exhibit significantly increased odds for depression [24,25]. Thus, it remains unclear whether children with CP present depressive symptoms. To answer this question, it is necessary to investigate the depressive symptoms using a self-rated questionnaire. 

Thus, the primary objective of the present study was to examine the difference in depressive symptoms between children with and without CP. We hypothesized that children with CP would exhibit a trend toward higher severity of depressive symptoms when compared with children without CP. The secondary objective was to examine how elevated depressive symptoms in children with and without CP are associated with age, severity of CP, and behavioral problems assessed by their parents. We hypothesized that some internalizing behavior individually accounts for the increased severity of some of the depressive symptoms in children with or without CP. Among mental health-related disorders, we focused on depression, as it directly affects the QOL. It is important to know the factors causing depressive symptoms in patients with CP for better planning to support their daily activities. 

## 2. Materials and Methods

### 2.1. Participants

Participants included 24 children with CP (CP group) and a comparison group of 33 TD children (TD group). The mean chronological age of the CP group was 12.3 ± 4.0 years (range: 6–18 years, 9 girls). The mean chronological age of the TD group was 12.2 ± 3.3 years (range: 6–17 years, 18 girls). The inclusion criteria for the CP group were (1) children with a formal diagnosis of CP, (2) children having a degree of motor impairment corresponding to levels I, II, III, and IV of the Gross Motor Function Classification System (GMFCS), (3) children having effective and clear verbal responses such as “yes” or “no”, and (4) children going to school almost every weekday. Exclusion criteria were presence of severe visual or auditory disability and lack of an ability to understand instructions regarding answering the questionnaire. No significant differences were observed in age (Mann–Whitney U = 388, *p* = 0.90) and sex ratio (chi-squared test *χ*^2^(1) = 1.47, *p* = 0.23) between the groups.

The parents provided written informed consent for participation of their children in this study. The study was conducted in accordance with the guidelines of the Declaration of Helsinki and was approved by the ethics committee of Japan Baptist Hospital (approval number: No. 17-6) and Beppu Developmental Medical Center (approval number: No. 29-1). 

### 2.2. Measures

#### 2.2.1. Depressive Symptoms

Depressive symptoms were assessed using the Birleson Depression Self-Rating Scale for Children (DSRS-C) [26]. The DSRS-C is a self-report questionnaire that consists of 18 items. It is widely used to measure depressive symptoms in children. The Japanese version of the DSRS-C prepared by Murata et al. has been confirmed to be reliable and valid [27]. 

Respondents were asked to rate each item on a 3-point scale ranging from 0 (never) to 2 (always). The total DSRS-C score ranges from 0 to 36, with higher scores indicating greater severity of depressive symptoms. Two subscales, namely ‘‘depressive mood’’ (9-items scale, total score 18) and ‘‘decline of activity and enjoyment’’ (9-items scale, total score 18) were extracted from the DSRS-C according to previous Japanese studies with large sample sizes [28,29]. The reliability of each factor was evaluated by calculating the reliability coefficient (Cronbach’s α), which was 0.79 for ‘‘decline of activity and enjoyment’’, 0.80 for ‘‘depressive mood’’, and 0.84 for DSRS-C total score [28]. These values are large enough to ensure the reliability of DSRS-C, which consists of two factors. 

Data were collected in a quiet room for the TD children at their home and for CP children at the child’s center for rehabilitation. In conducting the assessment, to avoid confusion among the children, we asked the participants to answer the questions in a similar manner employed by an examiner reading the questions in their home or clinical setting. We used the total DSRS-C score as an indicator of depressive symptoms and scores from the two subscales to identify the two different aspects that contribute to depressive symptoms. 

#### 2.2.2. Behavioral Assessment

Parents completed the Japanese version of the Strength and Difficulties Questionnaire (SDQ). The SDQ is one of the most widely used screening tools for psychopathology in children and adolescents [30]. This questionnaire comprises of 25 items across four difficulty subscales (hyperactivity/inattention, emotional symptoms, conduct problems, and peer problems) and one strength subscale, namely the prosocial behavior. Each subscale consists of five items. “Hyperactivity/inattention” and “conduct problems” are externalizing behaviors, while “emotional symptoms” and “peer problems” are internalizing behaviors. Parents rated each item using a 3-point scale (0: not true, 1: somewhat true, 2: certainly true). Total difficulty score (TDS) was also calculated by summation of the scores from the four difficulty subscales. The TDS ranged from 0 to 40. The Japanese version of SDQ has been shown to have good reliability (α = 0.81) and validity estimates [31,32]. Scores from subscale and TDS were used in the subsequent analysis.

### 2.3. Data Analysis

Differences in age and sex ratio between the groups were examined using Mann–Whitney U test and chi-squared test. For group comparisons of DSRS-S total and subscale scores, TDS, and scores from the SDQ subscales, we used Brunner–Munzel test, which does not require normality and equal variance of the data.

To investigate the association between self-rated depressive symptoms and behavioral problems assessed by parents, Spearman’s correlation coefficients were calculated. Multiple linear regression analyses were conducted with age, GMFCS (only the CP group), and scores from the five SDQ subscales as independent variables and DSRS-C scores as dependent variables for each group. 

A *p*-value < 0.05 was considered statistically significant. Statistical analysis was performed using the Jamovi project (2019) computer software (version 0.9, Sydney, Australia) [33] and R version 3.4.1 (R foundation, Vienna, Austria) [34].

## 3. Results

Table 1 shows the means and the standard deviations of the observed variables. The children with CP who participated in this study included eight children with GMFCS level I, four with level II, five with level III, and seven with level IV.

### 3.1. Group Comparisons of DSRS-C and SDQ

**Figure 1:**
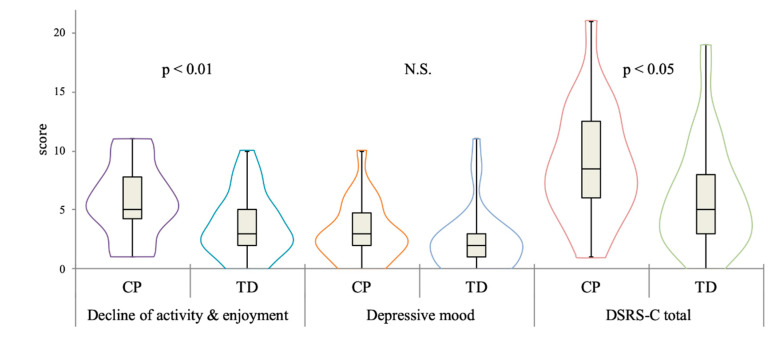
Comparison of DSRS-C scores between the groups.

Significant differences were observed in the “decline of activity and enjoyment’’ score (Brunner–Munzel Test Statistic = 2.62, *p* < 0.05) and the total DSRS-C score (Brunner–Munzel Test Statistic = 2.41, *p* < 0.05) between the groups. However, no significant difference was observed in the ‘‘depressive mood’’ score (Figure 1). In the SDQ comparison, scores from all difficulty subscales and TDS in the CP group were higher than those in the TD group (*ps* < 0.01), while prosocial behavior subscale scores were not significantly different between the groups. 

### 3.2. Correlation Analysis between Depressive Symptoms and Behavioral Features

In the CP group, age was not correlated with depression scores. However, it was negatively correlated with “conduct problems” (*ρ* = −0.51, *p* = 0.01), “hyperactivity/inattention” (*ρ* = −0.50, *p* = 0.01), and TDS (*ρ* = −0.47, *p* < 0.05) (Table 2). These findings indicate that externalizing behavioral problems in children with CP decrease with age, while the depressive symptoms do not change with age. Furthermore, the GMFCS level, which is an indicator of mobility functions, was not related to the depressive symptoms. In the correlation analysis between depressive symptoms and behavioral features, the “hyperactivity/inattention” score was negatively correlated with the “decline of activity and enjoyment” score. 

In the TD group, age was positively correlated with the “decline of activity and enjoyment” score (*ρ* = 0.52, *p* < 0.01) and the DSRS-C total score (*ρ* = 0.42, *p* < 0.05). Age was negatively correlated with the “hyperactivity/inattention” score (*ρ* = −0.37, *p* < 0.05) (Table 3). In additional correlation analysis between depressive symptoms and behavioral features, the “peer problems” score was positively correlated with the “decline of activity and enjoyment” score (*ρ* = 0.41, *p* < 0.05) and the DSRS-C total score (*ρ* = 0.48, *p* < 0.01). The “emotional symptoms” score was positively correlated with the “depressive mood” score (*ρ* = 0.39, *p* < 0.05) (Table 3). These findings suggested that depressive symptoms in TD children increased with age and the severity of depressive symptoms increased with increasing severity of internalizing behaviors. Thus, different factors were associated with depressive symptoms in the CP group and in the TD group.

### 3.3. Hierarchical Regressions Analysis to Identify the Factors Contributing to Depressive Symptoms

Separate hierarchical regression analyses for each dependent variable (the “decline of activity and enjoyment” score and the DSRS-C total score) were conducted to evaluate the confounding factors and the effect of behavioral features in daily life. The results of this analysis are presented in Table 4. There was a significant positive correlation between age and “decline of activity and enjoyment” score (*β* = 0.28), and significant group effect (*β* = 0.35) in step 1. However, when behavioral features were introduced in step 2, these significant correlations disappeared. Similarly, for the DSRS-C total score, when behavioral features were added as independent variables to the regression model in step 2, the significant group effect observed in step 1 disappeared. These results suggest that group effects were mediated by behavioral problems in daily life rather than by existence of motor deficits. Both the models showed that “hyperactivity/inattention” (*β* = −0.50 and −0.44) and “peer problems” (*β* = 0.38 and 0.40) as perceived by the parents were significant predictors of depressive symptoms (Table 4). Thus, low “hyperactivity/inattention” score and high “peer problems” scores were associated with greater severity of depressive symptoms in children.

## 4. Discussion

In the present study, we observed that self-rated depressive symptoms in children with CP were more severe than those in TD children. Particularly, decline of activities and enjoyment in their daily life was identified as a contributing factor to greater severity of depressive symptoms. In addition, the “decline of activity and enjoyment” score in children with CP was negatively correlated with the “hyperactivity/inattention” score, while depressive symptoms in TD children were positively correlated with age and the “peer problems” score. Hierarchical multiple regression analysis also revealed that the greater severity of depressive symptoms observed in children with CP was mediated by hyperactivity/inattention and peer problems rather than the effects of motor impairments. 

Recent studies have shown that children with CP have a higher prevalence of depression, which can be attributed to pain and low amount of physical activity [24]. Children with CP also exhibit less participation and enjoyment of social and recreational activities when compared with TD children [35,36]. Our results are consistent with the results from previous reports and suggest that the greater severity of depressive symptoms in children with CP is related to the inability to enjoy the activities of daily life. Several systematic reviews and meta-analyses have concluded that maintaining physical activity is an effective mode of intervention to prevent the onset of depression and to reduce depressive symptoms in children and youth [37,38,39,40,41]. Therefore, to prevent secondary mental health problems in children with CP, it may be necessary to create an environment or select an intervention aimed at enabling the children to enjoy the activities of daily living.

We observed that severity of CP and age were not associated with depressive symptoms. GMFCS level is an evaluation that focuses on the mobility of people with CP. It is not necessarily an indicator of social participation or the level of activity, which may explain the results in the present study. Indeed, it has been reported that physical activity in non-ambulatory toddlers with CP was not related to gross motor function [42]. On the other hand, depressive symptoms in the TD group increased with age. This finding is consistent with the results from a previous report that investigated the prevalence of depressive symptoms in children and adolescents from the general Japanese population using the DSRS-C [43]. Moreover, the significant correlation between depressive symptoms and peer problems observed in TD children has been reported in previous studies as well [44,45]. 

Interestingly, we found a negative correlation between depressive symptoms and the “hyperactivity/inattention” scores in the CP group. It is difficult to interpret this result, but it may be explained by the fact that inactivity of the depressed children has appeared calmer to the parents. Alternatively, it is possible that children with CP who showed high levels of activity might be perceived as hyperactive by their parents. There is room for a variety of interpretations on this issue, and further studies are needed to examine our findings in a larger population. 

The results from the hierarchical multiple regression analysis showed that the effects of having CP or age on depressive symptoms disappeared when the behavioral problems denoted by “hyperactivity/inattention” scores and “peer problems” scores were added to the regression models in step 2. Hyperactivity/inattention had a negative effect on depressive symptoms in children. As mentioned above, the characteristics of hyperactivity may be linked to the participation of children in activities outside home. However, the SDQ assessment does not separate hyperactivity and inattention. Hence, it is difficult to determine which of them had a relatively greater impact. In a study examining risk factors for the development of depression in children with attention-deficit/hyperactivity disorder, it was observed that inattention predicted the symptoms of depression via disruption of interpersonal functioning, while hyperactivity was not a predictive factor [46]. Similarly, it is possible that hyperactivity/inattention might have indirectly affected depression in the present study. In addition, we observed that peer problems had a significant impact on depressive symptoms in children with CP after controlling for age and gender. Earlier studies have shown that social problems such as peer problems are closely associated with depression in children with or without disabilities [24,42,45,47]. Our results have provided additional evidence that peer problems are related to depressive symptoms in children with CP. 

Notably, the decline of activity and enjoyment contributed to the higher DSRS-C total scores observed in the present study. Although the cause of inability to enjoy the activities remains unclear, rehabilitation therapists, supporters, and families need to provide opportunities for children with CP to actively participate in everyday activities from an early age. The opportunity for them to participate in such activities on a daily basis may help them maintain their motivation to move and enjoy these activities [48]. Moreover, participation in various activities may also promote the development of prosocial behavior and peer relationships. 

The present study has several limitations. We could not assess other factors such as presence or absence of pain and family environment that may cause depressive symptoms. Recently, it has been shown that presence of pain influences participation in physical leisure activities [49], which may lead to depressive symptoms. We did not find a significant relationship between the severity of CP and depressive symptoms. However, some studies have found a significant relationship between motor coordination problems and depressive symptoms in TD children [50,51,52]. These findings suggest that children with CP may also exhibit a relationship between mental health problems and difficulties in motor coordination using the upper limbs rather than mobility. Our measurements were limited to a cross-sectional assessment of only 24 participants with CP. Future studies with longitudinal measures in the course of development in a larger population would allow for more conclusive identification of the factors causing depression symptoms.

## 5. Conclusions

To the best of our knowledge, this is the first study that demonstrated that self-rated depressive symptoms in children with CP were more severe than those in TD children. Our results indicated that depressive symptoms in children with CP may be a precursor to the previously observed fact that adults with CP have a higher prevalence of depression. Particularly, decline in activities and enjoyment was identified as a contributor to the increased severity of depressive symptoms. Moreover, the greater severity of depressive symptoms in children with CP was mediated by hyperactivity/inattention and peer problems. Thus, considering the factors such as peer problems that may cause depressive symptoms, it is imperative to provide opportunities from an early age so that children with CP can actively participate in social activities.

## Figures and Tables

**Table 1:** Means and standard deviations (SD) of study variables.

Variables	CP Group (n = 24)		TD Group (n = 33)		*p* Value
	Mean	SD	Mean	SD	
Age (year)	12.2	4.0	12.2	3.3	*p* = 0.90
*DSRS-C*					
Decline of activity & enjoyment	5.71	2.99	3.76	2.67	*p* = 0.005
Depressive mood	3.17	2.46	2.52	2.62	*p* = 0.20
total score	8.88	4.71	6.27	4.51	*p* = 0.02
*SDQ*					
Conduct problems	2.67	2.20	1.33	1.34	*p* = 0.02
Hyperactivity/Inattention	3.88	2.71	2.18	2.19	*p* = 0.01
Emotional symptoms	2.01	1.91	0.67	0.85	*p* = 0.01
Peer problems	3.29	2.10	1.06	1.30	*p* < 0.01
Pro-social behavior	6.75	2.29	7.24	1.86	*p* = 0.56
TDS	11.91	6.19	5.24	3.91	*p* < 0.01

CP: cerebral palsy; TD: typically developing; DSRS-C: Depression Self-Rating Scale for Children; SDQ: Strengths and Difficulties Questionnaire; TDS: total difficulty score.

**Table 2:** Correlations between DSRS-C scores and age, mobility level and parent reported behavioral features in the CP group.

Participants with CP	Age	DSRS-C		
		Decline of Activity and Enjoyment	Depressive Mood	DSRS-C Total Score
Age	-	0.02	−0.32	−0.13
GMFCS level	-	−0.23	−0.17	−0.22
Conduct problems	−0.51 *	−0.06	0.20	0.07
Hyperactivity/Inattention	−0.50 *	−0.55 **	−0.03	−0.37
Emotional symptoms	−0.13	0.13	−0.03	0.10
Peer problems	−0.01	0.12	0.10	0.15
Pro-social behavior	0.40	0.39	−0.12	0.18
TDS	−0.47 *	−0.15	0.12	−0.02

* *p* < 0.05, ** *p* < 0.01. GMFCS: Gross Motor Function Classification System.

**Table 3:** Correlations between DSRS-C scores and age and parent reported behavioral features in the TD group.

Participants with TD	Age	DSRS-C		
		Decline of Activity and Enjoyment	Depressive Mood	DSRS-C Total Score
Age	-	0.52 **	0.22	0.42 *
Conduct problem	0.01	0.13	0.20	0.22
Hyperactivity/Inattention	−0.37 *	−0.34	−0.21	−0.29
Emotional symptoms	−0.13	0.17	0.39 *	0.34
Peer problem	−0.05	0.41 *	0.33	0.48 **
Pro-social behavior	−0.10	−0.11	0.21	−0.03
TDS	−0.19	0.12	0.13	0.19

* *p* < 0.05, ** *p* < 0.01.

**Table 4:** Hierarchical regression analyses predicting depressive symptoms.

**Decline of Activity and Enjoyment**	**B**	**SE**	***β***	***t***	***p***	***R^2^***	***⊿*** ***R^2^***	***F***
*Step 1*						0.21	-	4.72
Age	0.23	0.10	0.28	2.27	0.03 *			
Gender (Female-Male)	0.73	0.73	0.13	1.01	0.32			
Group (CP-TD)	2.06	0.73	0.35	2.81	0.01 **			
*Step 2*						0.48	0.27 **	5.56
Age	0.15	0.01	0.18	1.52	0.14			
Gender	0.22	0.66	0.04	0.34	0.74			
Group	1.01	0.81	0.17	1.25	0.22			
Conduct problem	0.23	0.21	0.14	1.05	0.30			
Hyperactivity/inattention	−0.58	0.16	−0.50	−3.60	<0.01 **			
Emotional symptoms	0.31	0.24	0.16	1.29	0.20			
Peer problem	0.57	0.21	0.38	2.65	0.01 **			
Pro-social behavior	0.11	0.18	0.08	0.61	0.55			
**DSRS-C Total Score**	**B**	**SE**	***β***	***t***	***p***	***R^2^***	***⊿*** ***R^2^***	***F***
*Step 1*						0.12	-	2.43
Age	0.22	0.17	0.16	1.27	0.21			
Gender (Female-Male)	1.12	1.24	0.12	0.91	0.37			
Group (CP-TD)	2.78	1.24	0.29	2.24	0.03 *			
*Step 2*						0.40	0.28 **	4.00
Age	0.16	0.17	0.12	0.93	0.36			
Gender	0.30	1.14	0.03	0.26	0.79			
Group	0.63	1.40	0.07	0.45	0.66			
Conduct problem	0.72	0.37	0.28	1.93	0.06			
Hyperactivity/inattention	−0.83	0.28	−0.44	−2.98	0.01 **			
Emotional symptoms	0.33	0.42	0.11	0.80	0.43			
Peer problem	0.95	0.37	0.40	2.58	0.01 **			
Pro-social behavior	0.25	0.31	0.11	0.82	0.42			

B: Partial regression coefficient, SE: standard error, *β*: standardized regression coefficient, *t*: Student’s t-distribution, *R^2^*: determination coefficient, F: Scedecor’s F-distribution, * *p* < 0.05, ** *p* < 0.01.
